# A one-step fluorescent biosensing strategy for highly sensitive detection of HIV-related DNA based on strand displacement amplification and DNAzymes†

**DOI:** 10.1039/c8ra06480f

**Published:** 2018-09-12

**Authors:** Xiaoyu Yan, Min Tang, Jianru Yang, Wei Diao, Hongmin Ma, Wenbin Cheng, Haiying Que, Tong Wang, Yurong Yan

**Affiliations:** Key Laboratory of Clinical Laboratory Diagnostics (Ministry of Education), College of Laboratory Medicine, Chongqing Medical University Chongqing 400016 China yanyurong163@163.com +86-23-684852 +86-23-684852; Department of Clinical Laboratory, Affiliated Hospital of Zunyi Medical University Zunyi 563003 China

## Abstract

Sensitive and specific detection of HIV-related DNA is of great importance for early accurate diagnosis and therapy of HIV-infected patients. Here, we developed a one-step and rapid fluorescence strategy for HIV-related DNA detection based on strand displacement amplification and a Mg^2+^-dependent DNAzyme reaction. In the presence of target HIV DNA, it can hybridize with template DNA and activate strand displacement amplification to generate numerous DNAzyme sequences. With the introduction of Mg^2+^, DNAzyme can be activated to circularly cleave the substrate DNA, which leads to the separation of fluorophore reporters from the quenchers, resulting in the recovery of the fluorescence. Under the optimal experimental conditions, the established biosensing method can detect target DNA down to 61 fM with a linear range from 100 fM to 1 nM, and discriminate target DNA from mismatched DNA perfectly. In addition, the developed biosensing strategy was successfully applied to assay target DNA spiked into human serum samples. With the advantages of fast, easy operation and high-performance, this biosensing strategy might be an alternative tool for clinical diagnosis of HIV infection.

## Abstract 2

A one-step fluorescent biosensing strategy for highly sensitive detection of HIV-related DNA based on strand displacement amplification and Mg^2+^-dependent DNAzyme reaction.

## Graphical abstract

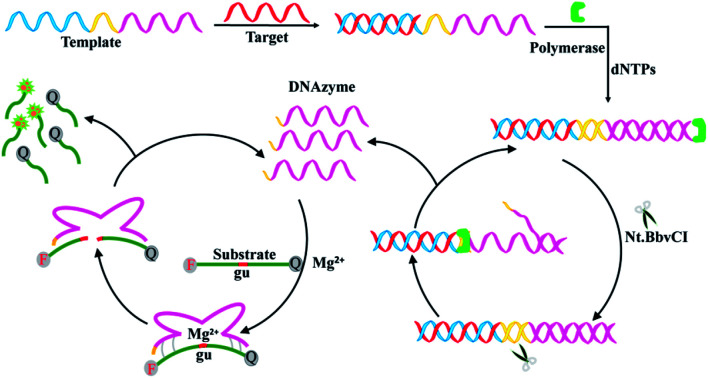

## 1. Introduction

Human immunodeficiency virus (HIV), a retrovirus, can lead to acquired immunodeficiency syndrome (AIDS) which destroys the infected patient's immune system.^1^ There are currently 36.7 million people living with HIV worldwide, but almost 60% of infected adults don't have access to antiretroviral treatment mostly due to the failure of early diagnosis.^2^ It is reported that the mortality rate of AIDS has decreased significantly with the wide application of combined antiretroviral therapy.^3^ Therefore, early diagnosis and therapy of HIV infection can effectively prevent HIV transmission and prolong survival period.^4^

Immunological methods such as enzyme-linked immunosorbent assay (ELISA) and western blot (WB) are common methods for clinical diagnosis of HIV infection.^5,6^ However, it takes a period of time for the body to produce the corresponding antibodies after infection with the HIV virus. Therefore, there is a “window period” for HIV detection, which makes the early diagnosis of HIV infection full of challenges.^7,8^

In recent years, nucleic acid assays have attracted increasing attention because they can prompt for viral infection even in the absence of HIV antibodies.^9^ With high sensitivity and specificity, polymerase chain reaction (PCR) has evolved as the most commonly used technique for nucleic acid detection. Nevertheless, it is not suitable for short-length DNA detection due to the complexity in primer design.^10^ Moreover, PCR-based methods need thermal cycler and specialized person which limit their use in resource-poor and impoverished areas. Thus, it is highly desirable to develop universal and cost-effective approaches for sensitive and specific detection of HIV-related DNA.

So far, various biosensing methods have thrown light on HIV-related DNA detection, such as colorimetry,^11^ electrochemistry,^12^ surface plasmon resonance (SPR),^13^ and fluorescence resonance energy transfer (FRET) *etc.*^14^ Among these methods, fluorescent method usually takes precedence as an alternative platform owing to its advantages of high sensitivity and simple procedure. To further improve the performance of the biosensors, different isothermal amplification strategies have been developed for genetic test and medical diagnosis, such as rolling circle amplification (RCA),^15–18^ hybridization chain reaction (HCR),^19,20^ catalytic hairpin assembly (CHA),^21,22^ exonuclease III aided signal amplification,^23,24^ and strand displacement amplification (SDA).^25^ Combining polymerase-mediated strand extension with nicking enzyme-assisted single strand cleaving process, SDA is an isothermal molecular chain reaction with high amplification efficiency. Due to the DNA biological circuit with feedback design, the target DNA switches to exponentially generate numerous secondary DNA molecules. Herein, SDA-based signal amplification techniques show great potential for highly sensitive and effective determination of target DNA.^26^ When coupled with other signal amplification strategies, it can be applied to construct versatile biosensing system.

DNAzyme, as an alternative tool enzyme to protein enzyme or ribozyme, is a kind of functional catalytic nucleic acid sequence selected by systematic evolution of ligands by exponential enrichment (SELEX) *in vitro*.^27^ Horseradish peroxidase (HRP) mimicking DNAzyme possesses a special G-quadruplex structure which exhibited peroxidase-like activity after binding to hemin and catalyzed to generate a colorimetric or chemiluminescent signal.^28–30^ Although these colorimetric or chemiluminescent approaches based on HRP-mimicking DNAzyme are well-established, they usually involve complicated operation process, long assay time and insufficient sensitivity. Differing from the HRP-mimicking DNAzyme, metal ion-dependent DNAzyme is a class of RNA-cleaving DNAzyme which can catalyze the cleavage of RNA substrates or the ribonucleotides embedded in a chimeric DNA substrate with metal ion as cofactors.^31^ Due to the inherent advantages of significant catalytic efficiency, excellent biocompatibility and good stability, metal ion-dependent DNAzyme is much more amenable to combing with other signal amplification strategies and has attracted substantial research in various detection platforms. Particularly, because of its cyclic cleavage property, the metal ion-dependent DNAzyme can also be applied as signal amplification unit.^32–34^ Thus, metal ion-dependent DNAzyme is frequently combined with other signal amplification strategies^35,36^ to further improve the detection performance of constructed biosensors.

Herein, a facile, rapid and one-step homogeneous fluorescence strategy was developed for HIV-related DNA detection by integrating SDA with Mg^2+^-dependent DNAzyme catalytic reaction. The proposed method exhibited good performance for HIV-related DNA detection with high sensitivity and excellent mismatch distinguishing ability. More importantly, the combination of SDA signal amplification and Mg^2+^-dependent DNAzyme catalytic recycling in our strategy ensures the entire reaction to be performed by one-step operation and reduce the detection time to 60 min. In addition, it could also be applied to assay the concentration of target HIV DNA spiked into human serum samples. Therefore, the proposed fluorescence biosensing strategy presents an excellent platform towards HIV-related DNA analysis, which has great application potential for biomedical research and clinical diagnosis of HIV infection.

## 2. Experiment section

### 2.1. Materials and reagents

All oligonucleotide sequences were synthesized and purchased from Sangon Inc. (Shanghai, China). Table S1 in ESI† shows the sequences of the oligonucleotides used in the study. All oligonucleotides were HPLC-purified and dissolved in tris-ethylenediaminetetraacetic acid (TE) buffer (pH 8.0, 10 mM Tris–HCl, 1 mM ethylene diamine tetraacetic acid) and stored at −20 °C, which were diluted in appropriate buffer prior to use. DL20 DNA marker was purchased from Takara (Dalian, China). Gold view (GV) was purchased from SBS Genetech (Beijing, China). Klenow fragment and Nb.BbvCI nicking enzyme were purchased from New England Biolabs Inc. (Beverly, MA, USA). Deoxynucleotide triphosphates (dNTPs) were obtained from Sangon Inc. (Shanghai, China). All other chemicals not mentioned here were of analytical reagent grade. Millipore-Q water (≥18 MΩ) was used in all experiments. Human serum samples were obtained from the First Affiliated Hospital of Chongqing Medical University.

### 2.2. Isothermal amplification system

The strand displacement amplification and DNAzyme cleavage reaction were simultaneously performed in one-step. The reaction mixture contained 0.5 μM template, 0.25 μM fluorophore/quencher-functionalized substrates, various concentrations of target DNA, 0.5 mM dNTPs, 5 unit Nb.BbvCI nicking enzyme and 3 unit Klenow fragment in 1× CutSmart™ buffer (20 mM pH 7.9 Tris-acetate, 500 mM potassium acetate, 10 mM magnesium acetate, 100 μg mL^−1^ BSA) as well as 1× Klenow fragment buffer (100 mM pH 7.5 Tris–HCl, 70 mM MgCl_2_, 1 mM DTT) to yield a final volume of 20 μL, then the mixture was incubated at 37 °C for 60 min.

### 2.3. Fluorescence measurement

After the strand displacement amplification and DNAzyme cleavage reaction, 80 μL H_2_O was added to the mixture to yield a final volume of 100 μL. Then, all fluorescence measurements were performed on a Cary Eclipse Fluorescence spectrophotometer (Agilent, California), using a quartz fluorescence cell with an optical path length of 1.0 cm. The fluorescence emission spectra were recorded in the region from 500 to 600 nm in a quartz cuvette at an excitation wavelength of 492 nm. The maximum fluorescence emission intensity was obtained at 518 nm. Both the excitation and emission slit widths were set at 5 nm at room temperature. Prior to each experiment, all cuvettes were washed with 70% ethanol and distilled water.

### 2.4. Gel electrophoresis

The feasibility of the SDA reaction was explored by 8% native polyacrylamide gel electrophoresis (PAGE), which was conducted on DYY-6C electrophoresis analyzer (Liuyi Instrument Company, China) in 1× TBE buffer (90 mM Tris–HCL, 90 mM boric acid, 2 mM EDTA, pH 7.9) at a 120 V constant voltage for 30 min. The gel was stained with gold view (GV) for 30 min and photographed by gel imaging analysis system (Bio-Rad, USA).

### 2.5. Human serum samples preparation

The human serum samples were diluted 25 times prior to detection. Then, the target DNA was detected in these human blood plasma samples following the same procedure.

## 3. Results and discussion

### 3.1. Principle of the fluorescence assay

**Fig. 1:**
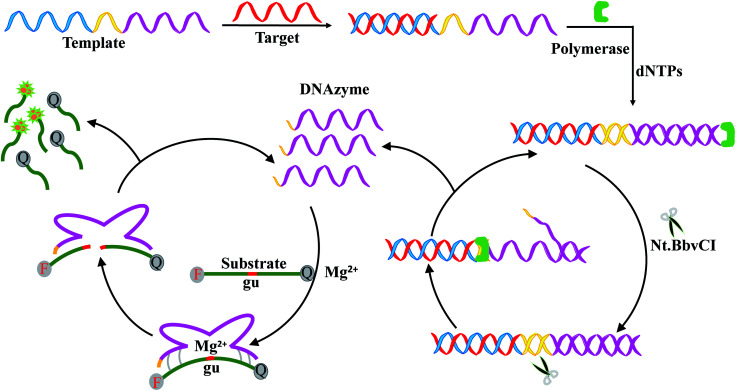
Schematic representation of HIV DNA detection based on strand displacement reaction and DNAzyme reaction.

In this work, a signal-on fluorescence method was developed for HIV-related DNA detection based on strand displacement amplification and Mg^2+^-dependent DNAzyme catalytic reaction. Fig. 1 shows the working principle. The system includes templates, ribonucleobase-containing substrate DNA functionalized with a fluorophore/quencher pair, Nb.BbvCI nicking enzyme, Klenow fragment, deoxynucleotide solution mixture (dNTPs) and Mg^2+^ ions. The template consists of two nucleic acid scaffolds that include recognition site for the target DNA and replication track that yields the nicking domain for Nb.BbvCI and Mg^2+^-dependent DNAzyme sequence. In the presence of target DNA, the specific hybridization between target DNA and the corresponding domain of the template forms a partial duplex. Then, along the template the target DNA is extended to form a complete duplex with the help of Klenow fragment and dNTPs. Subsequently, the nicking enzyme can specifically recognize the newly formed duplex nicking site, cleaving the upper extended DNA strand and exposing a new replication site for polymerase. This results in the secondary replication of the strand, while displacing the DNAzyme sequence. Thus, a large number of DNAzyme sequences are produced through the continuously extension, cleavage and strand displacement amplification. After introduction of cofactor Mg^2+^ ions, DNAzyme sequence can be activated to circularly cleave the fluorophore/quencher-functionalized nucleic-acid substrates. After cleavage, fluorophore/quencher pairs of substrates are separated from each other, resulting in great amplification fluorescence signal.

### 3.2. Feasibility assay of the sensing strategy

**Fig. 2:**
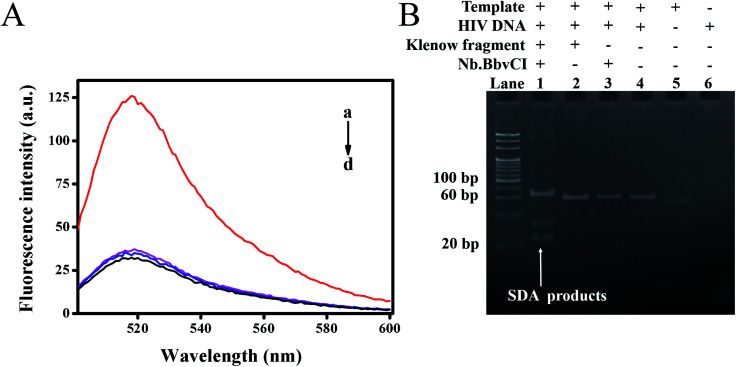
(A) Fluorescence signal curves of different mixtures: (a) Template + HIV DNA + Klenow fragment polymerase + Nb.BbvCI; (b) Template + HIV DNA + Klenow fragment polymerase; (c) Template + HIV DNA + Nb.BbvCI; (d) Template + Klenow fragment polymerase + Nb.BbvCI. The concentration of target HIV DNA is 1 nM. (B) Native PAGE analysis: M: DNA marker; lane 1: SDA; lane 2: Template + HIV DNA + Klenow fragment polymerase; lane 3: Template + HIV DNA + Nb.BbvCI; lane 4: Template + HIV DNA; lane 5: Template; lane 6: HIV DNA.

To verify whether the target DNA could initiate the reaction, leading to a detectable fluorescence signal, we explored the feasibility of this proposed method through comparative experiments, in which fluorescence emission spectra of different mixtures were recorded. As shown in Fig. 2A, the mixture without target DNA showed very weak fluorescence intensity at 518 nm due to the efficient quenching of FAM by the closely positioned BHQ-1 (curve d). The control experiment without Klenow fragment (curve b) or Nb.BbvCI (curve c) also exhibited weak fluorescence intensity, indicating that exclusion of either the nicking endonuclease or polymerase could prohibit the strand displacement amplification. On the contrary, the fluorescence intensity had significant enhancement upon the addition of target DNA to the mixture containing both the Klenow fragment and Nb.BbvCI (curve a). Thus, the cooperative polymerization by the polymerase and scission by the nicking enzyme were indispensable to self-assemble the DNAzyme sequences.

Furthermore, a native polyacrylamide gel electrophoresis (PAGE) was also carried out to verify the strand displacement reaction products. As shown in Fig. 2B, the newly produced band, representing the products of SDA, was observed with the addition of target DNA (lane 1). However, the corresponding products could not be observed in the control group without the addition of Nb.BbvCI (lane 2) or Klenow fragment (lane 3). These results were in line with our previous fluorescence results. Consequently, our results demonstrated that SDA could be initiated in the presence of target HIV DNA and effectively generated a large number of DNAzyme sequences.

### 3.3. Optimization of experiment conditions

**Fig. 3:**
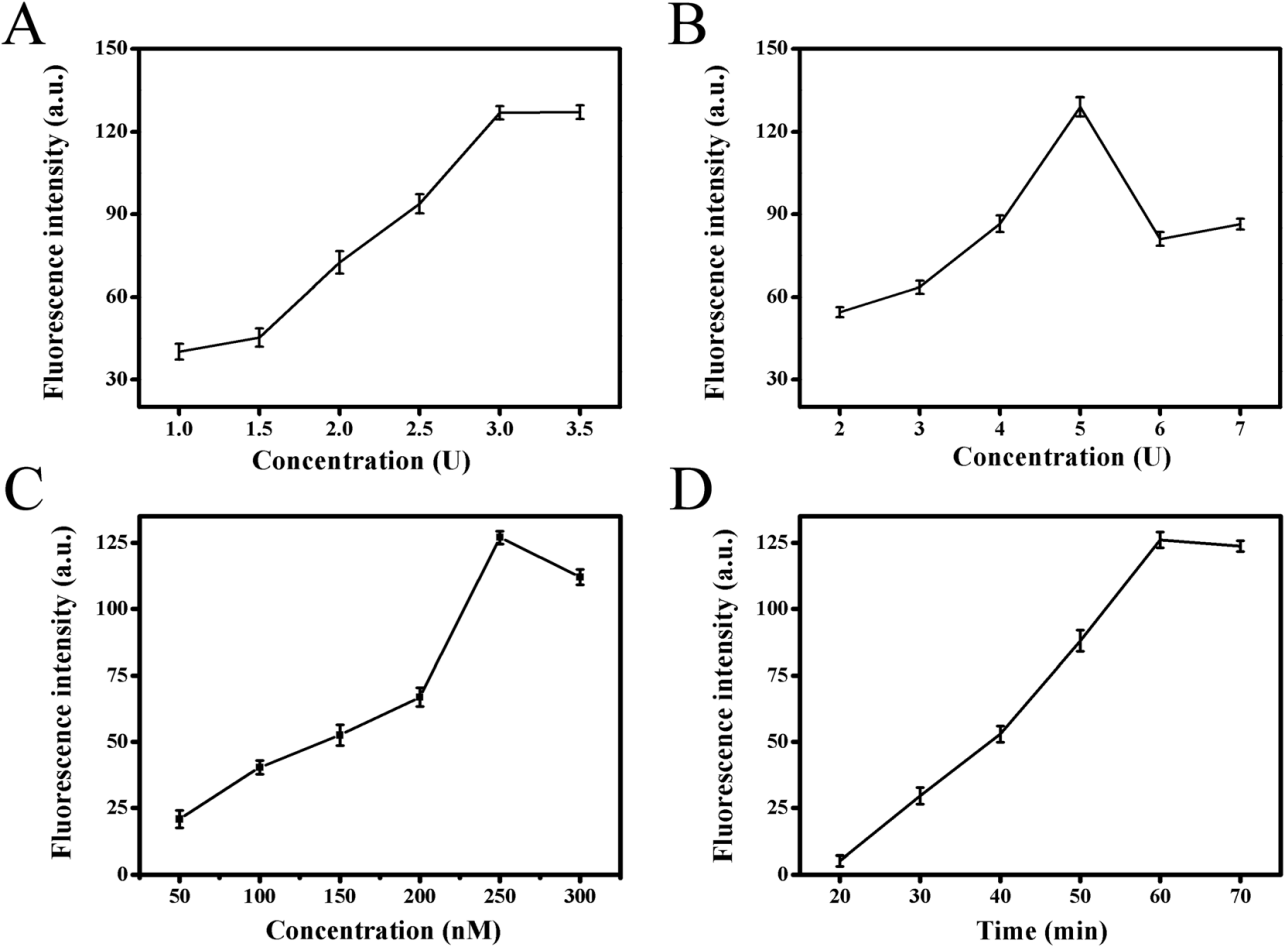
Optimizations of experimental parameters: (A) evaluation of the effect of Klenow fragment polymerase concentration, (B) evaluation of the effect of concentration of Nb.BbvCI, (C) evaluation of the effect of substrate concentration, (D) evaluation of the effect of the total reaction time. When one parameter changed, the others were under their optimal conditions. The error bars represented the standard deviations in three different measurements for each concentration.

In order to obtain the best analytical performance of the proposed strategy, several parameters were optimized, such as Klenow fragment concentration, Nb.BbvCI nicking enzyme amount, substrate concentration and total reaction time (Fig. 3). The fluorescence intensity was used to assess the performance. Firstly, it was clear that strand displacement amplification played crucial role for high sensitivity detection of target in this strategy. Therefore, the two key factors of Klenow fragment and Nb.BbvCI nicking endonuclease concentration in this process were optimized. Fig. 3A presented the effect of Klenow fragment concentration on fluorescence response. With increasing polymerase concentration from 1.0 to 3.0 units, the fluorescence intensity increased gradually, and the fluorescence signal tended to be a steady value at 3.0 units. Therefore, 3.0 units were selected as the optimal polymerase concentration in the subsequent experiments. The effect of Nb.BbvCI nicking endonuclease concentration on fluorescence response was also investigated, and the results were illustrated in Fig. 3B. When the concentration of Nb.BbvCI varied from 2 to 5 units, the fluorescence intensity improved correspondingly, further enhancing Nb.BbvCI concentration, the fluorescence intensity tended to level off. Therefore, 5 unit was selected as the optimal concentration. Secondly, the influence of functionalized substrate concentrations was investigated. Fig. 3C showed the effect of the concentration of fluorophore/quencher-functionalized substrates. The fluorescence signal reached maximum when the concentration of substrates probe was 250 nM and decreased at higher concentrations. Therefore, we set the concentration of substrates as 250 nM throughout this work. Finally, the impact of the total reaction time was illustrated in Fig. 3D. As the reaction time was extended from 20 to 60 min, the fluorescence intensity increased sharply and reached maximum at 60 min, then it tended to decrease after 60 min. Consequently, 60 min was chosen as the optimal reaction time.

### 3.4. Analytical performance of the biosensing method

**Fig. 4:**
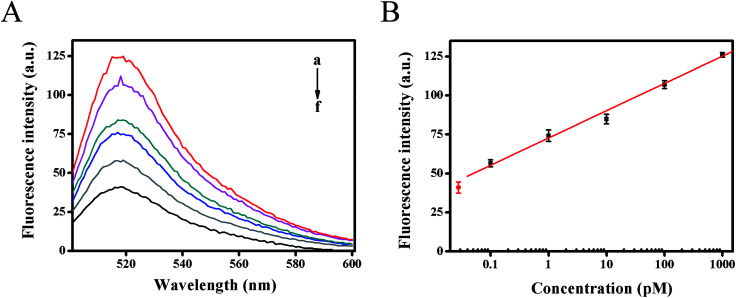
Dynamic range and sensitivity investigation by detecting HIV DNA at various concentrations: (a) 1 nM; (b) 100 pM; (c) 10 pM; (d) 1 pM; (e) 100 fM; (f) 0 pM. (A) Fluorescence emission spectra in a region of 500–600 nm. (B) Relationship of fluorescence signal peak (at 518 nm) with logarithm of target DNA. The error bars represented the standard deviations in three different measurements for each concentration.

The dynamic range and sensitivity of the proposed fluorescence strategy was confirmed under the optimal experimental conditions. As shown in Fig. 4A, the fluorescence intensity moved up with the increase of target HIV DNA concentration. In the range from 100 fM to 1 nM of target DNA concentration, the calibration plots demonstrated a good linear relationship between the fluorescence intensity (*F*) (at 518 nm) and the logarithm of target DNA concentrations (*C*) (Fig. 4B). The resulting linear equation was *F* = 17.4847 lg *C* (pM) + 72.7695 with a correlation coefficient of 0.9974 and detection limit of 61 fM from three times the standard deviation corresponding to the blank sample detection, which was much lower than previous reported methods for detecting HIV-related DNA (Table S2†). The high sensitivity of the developed strategy could be attributed to the high amplification efficiency of strand displacement amplification as well as the Mg^2+^-dependent DNAzyme catalytic reaction, providing an alternative detection strategy for HIV-related DNA.

### 3.5. Specificity and reproducibility of the biosensing method

**Fig. 5:**
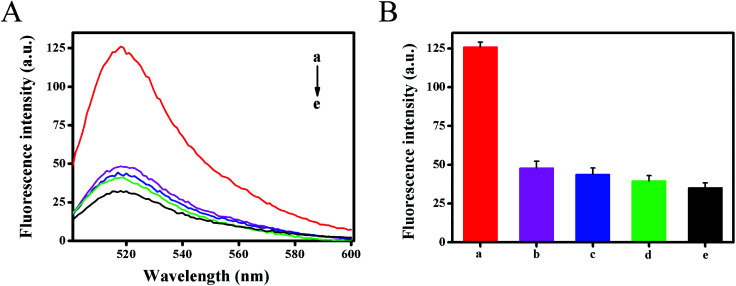
Specificity of this strategy for HIV DNA detection: (a) 1 nM of target DNA; (b) 1 nM of single-base mismatched strand (DNA-1); (c) 1 nM of double-base mismatched strand (DNA-2); (d) 1 nM of non-complementary mismatched strand (DNA-3); (e) the same reaction mixtures without HIV DNA were used as control. (A) Fluorescence emission spectra in a region of 500–600 nm. (B) Histogram of fluorescence signal peak (at 518 nm) for 5 types of samples.

To investigate the selectivity of the developed method, the fluorescence intensity for different targets (1 nM) were tested under the same experimental conditions, including target HIV-related DNA, single base mismatched DNA-1, two-base mismatched DNA-2 and non-complementary DNA-3 (Fig. 5). The results showed that DNA-3 (curve d) caused negligible changes in fluorescence signals against the blank test (curve e), the DNA-1 (curve b) and DNA-2 (curve c) caused only a slight increase. However, in the presence of HIV DNA, a dramatic increase in the fluorescence signal was obtained (curve a). It could be ascribed to the fact that only target HIV DNA could hybridize with template DNA with high efficiency and activate strand displacement amplification to generate abundant Mg^2+^-dependent DNAzyme for signal transduction. This result also demonstrated that the designed method had good selectivity and single nucleotide difference discrimination ability. Five replicate measurements of target HIV DNA at 1 pM and 100 pM showed the variation coefficients of 4.6% and 1.5%, respectively.

### 3.6. Detection of HIV-related DNA in human serum samples

To investigate the interference of biological samples on the fluorescence strategy, the proposed spiked assay was carried out by adding different concentrations (10 pM, 100 pM and 1 nM) HIV-related DNA to 25-fold-diluted serum sample. As shown in Table S3,† the recoveries for three detections were 92.5%, 115.7% and 97.7%, respectively, suggesting that complex mixtures have little effect on the detection performance. Thus, the established strategy could become a potential tool for HIV DNA assay in real biological samples.

## 4. Conclusion

In summary, we have successfully demonstrated a one-step and rapid fluorescence biosensing strategy for HIV-related DNA detection based on SDA and Mg^2+^-dependent DNAzyme catalytic reaction. Under the cyclic process of extension, cleavage and strand displacement reaction, low amount of target HIV-related DNA could generate lots of Mg^2+^-dependent DNAzyme sequences with excellent cyclic cleavage property, which greatly improved the sensitivity for detection of target DNA down to 61 fM. Even more noteworthy, the combination of SDA signal amplification and Mg^2+^-dependent DNAzyme catalytic recycling in our strategy ensures the entire detecting process to be performed by one-step operation and only takes 60 min to get an excellent detection fluorescence signal. Thus, the proposed fluorescence biosensing strategy has great application potential for clinical diagnosis of HIV infection. More importantly, this fluorescence method could be conveniently applied to other nucleic acids detection by adjusting the corresponding DNA sequences. Therefore, with one-step operating process and good analytical performance, the developed strategy offers great potential for point-of-care diagnostics of disease related DNA, miRNA as well as other biomarkers.

## Conflicts of interest

The authors declare no conflict of interest.

## Supplementary Material

RA-008-C8RA06480F-s001

## References

[cit1] Hanna D. B., Pfeiffer M. R., Torian L. V., Sackoff J. E. (2008). AIDS Patient Care STDS..

[cit2] Egger M., May M., Chêne G., Phillips A. N., Ledergerber B., Dabis F., Costagliola D., D'Arminio M. A., De W. F., Reiss P. (2002). Lancet.

[cit3] Cohen M. S., Chen Y. Q., Mccauley M., Gamble T., Hosseinipour M. C., Kumarasamy N., Hakim J. G., Kumwenda J., Grinsztejn B., Pilotto J. H. (2016). N. Engl. J. Med..

[cit4] Granich R. M., Gilks C. F., Dye C., Cock K. M. D., Williams B. G. (2009). Lancet.

[cit5] De l. R. R., Stevens M. M. (2012). Nat. Nanotechnol..

[cit6] Zhou L., Huang J., Yu B., Liu Y., You T. (2015). ACS Appl. Mater. Interfaces.

[cit7] Clerici M., Giorgi J. V., Chou C. C., Gudeman V. K., Zack J. A., Gupta P., Ho H. N., Nishanian P. G., Berzofsky J. A., Shearer G. M. (1992). J. Infect. Dis..

[cit8] Taylor D., Durigon M., Davis H., Archibald C., Konrad B., Coombs D., Gilbert M., Cook D., Krajden M., Wong T. (2015). Int. J. STD AIDS.

[cit9] Yin D., Tao Y., Tang L., Li W., Zhang Z., Li J., Xie G. (2017). Microchim. Acta.

[cit10] Véronique A. F., Marie-Laure C., Stéphane B., Marianne B., Corinne F., Kadidia T., Marie-Christine A., Josiane W., Christine R. (2010). J. Med. Virol..

[cit11] Long Y., Zhou C., Wang C., Cai H., Yin C., Yang Q., Dan X. (2016). Sci. Rep..

[cit12] Yijia W., Xiaoning B., Wei W., Xiuhua Z., Shengfu W. (2015). ACS Appl. Mater. Interfaces.

[cit13] Wu L., Zhang Q., Su L., Huang M., Zhao J., Yang M. (2007). Sens. Actuators, B.

[cit14] Qaddare S. H., Salimi A. (2017). Biosens. Bioelectron..

[cit15] Liu D., Daubendiek S. L., Zillman M. A., Ryan K., Kool E. T. (1996). J. Am. Chem. Soc..

[cit16] Zhang W., He Z., Yi L., Mao S., Li H., Lin J. M. (2017). Biosens. Bioelectron..

[cit17] Park K. W., Chang Y. L., Batule B. S., Park K. S., Park H. G. (2018). RSC Adv..

[cit18] Jiang H., Xu Y., Dai L., Liu X., Kong D. (2018). Sens. Actuators, B.

[cit19] Huang D. J., Huang Z. M., Xiao H. Y., Wu Z. K., Tang L. J., Jiang J. H. (2018). Chem. Sci..

[cit20] Bao B., Zhu J., Gong L., Chen J., Pan Y., Wang L. (2017). RSC Adv..

[cit21] Xu X., Wang L., Wu Y., Jiang W. (2017). Analyst.

[cit22] Zang Y., Lei J., Ling P., Ju H. (2015). Anal. Chem..

[cit23] Min X., Zhang M., Huang F., Lou X., Xia F. (2016). ACS Appl. Mater. Interfaces.

[cit24] Liu M. X., Liang S., Tang Y., Tian J., Zhao Y. C., Zhao S. (2018). RSC Adv..

[cit25] Zhang D. Y., Seelig G. (2011). Nat. Chem..

[cit26] Wang B., Wang X., Wei B., Huang F., Yao D., Liang H. (2017). Nanoscale.

[cit27] Ren K., Wu J., Ju H., Yan F. (2015). Anal. Chem..

[cit28] Li W., Liu Z., Lin H., Nie Z., Chen J., Xu X., Yao S. (2010). Anal. Chem..

[cit29] Weizmann Y., Beissenhirtz M. K., Cheglakov Z., Nowarski R., Kotler M., Willner I. (2010). Angew. Chem., Int. Ed..

[cit30] Li D., Wieckowska A., Willner I. (2010). Angew. Chem., Int. Ed..

[cit31] Peng H., Li X. F., Zhang H., Le X. C. (2017). Nat. Commun..

[cit32] Kim H. K., Rasnik I., Liu J., Ha T., Lu Y. (2007). Nat. Chem. Biol..

[cit33] Wu P., Hwang K., Lan T., Lu Y. (2013). J. Am. Chem. Soc..

[cit34] Freage L., Wang F., Orbach R., Willner I. (2014). Anal. Chem..

[cit35] Xu H., Xu Y., Bai L. (2015). Microchim. Acta.

[cit36] Yin H. S., Li B. C., Zhou Y. L., Wang H. Y., Wang M. H., Ai S. Y. (2017). Biosens. Bioelectron..

