# Supplementary material for: A one-step fluorescent biosensing strategy for highly sensitive detection of HIV-related DNA based on strand displacement amplification and DNAzymes
Source: RSC Adv. 2018 Sep 12;8(55):31710–6. doi: 10.1039/c8ra06480f (PMC9085900; doi:10.1039/c8ra06480f)
Supplement: RA-008-C8RA06480F-s001 [file RA-008-C8RA06480F-s001.pdf]

Electronic Supplementary Material (ESI) for RSC Advances.
This journal is © The Royal Society of Chemistry 2018

Supporting Information

A one-step fluorescent biosensing strategy for highly sensitive detection of HIV-related DNA based on strand displacement amplification and DNAzyme

Xiaoyu Yan [a, 1], Min Tang [a, 1], Jianru Yang [b], Wei Diao [a], Hongmin Ma [a], Wenbin Cheng [a], Haiying Que[a], Tong Wang [a], Yurong Yan [a,*]

[a] *Key Laboratory of Clinical Laboratory Diagnostics (Ministry of Education), College of Laboratory Medicine, Chongqing Medical University, Chongqing 400016, China.*

[b] *Department of clinical laboratory, Affiliated Hospital of Zunyi Medical University, Zunyi 563003, China.*

* Corresponding author. Tel: +86-23-68485240; Fax: +86-23-6848240.
E-mail address: yanyurong163@163.com (Y. Yan)

[1] These authors contributed equally to this work.

Table S1 DNA sequences used in this work

DNA	DNA sequence (from 5' to 3')
HIV	AGTCAGTGTGGAAAATCTCTAGC
DNA	TCAACCACATCGTTGTAGCTAGCCTGGCATCTCCTCAGC
Template	TAGAGATTTTCCACACTGACT
	AGTCAGTGTGGAAAATCTCAAGC
DNA-1	AGTCAGTGTGGAAAAACTCAAGC
DNA-2	AGGCACAAACACGCACCTCAAAG
DNA-3	FAM--TCAACCACAguGGCATCTG--BHQ-1
Substrate	

gu: ribonucleotide

Underline portion represents mutation base in target DNA.

Table S2 Comparison of different platforms for HIV-related DNA detection

Platform	Strategy [a]	Dynamic range	LOD	Reference
Fluorescence	T7 exonuclease	50 pM-8 nM	9.1 pM	1
Fluorescence	MB and Ag NCs	10 nM-200 nM	4.4 nM	2
Colorimetry	Glucose oxidase and CHA	10 pM-120 nM	4.8 pM	3
Electrochemical	triplex-forming molecular beacon	0.1 nM-10 nM	54 pM	4
Fluorescence	SDA and DNAzyme	0.1 pM-1 nM	61 fM	**This work**

[a] MB, molecular beacon; Ag NCs, silver nanoclusters; CHA, catalytic hairpin assembly; SDA, strand displacement amplification.

Table S3 Recovery results of HIV DNA spiked in human serum sample

samples	spiked (pM)	measured (pM)	relative error (%)	recovery (%)
1	10	9.251	3.5	92.5
2	100	115.721	4.1	115.7
3	1000	977.237	2.3	97.7

Reference:

1. L. Wang, J. Tian, Y. Huang, X. Lin, W. Yang, Y. Zhao, S. Zhao, *Microchim. Acta*, 2016, **183**, 2147-2153

2. Q. Cao, Y. Teng, X. Yang, J. Wang, E. Wang, *Biosens. Bioelectron.*, 2015, **74**, 318-321.

3. Y. Long, C. Zhou, C. Wang, H. Cai, C. Yin, Q. Yang and X. Dan, *Sci. Rep.*, 2016, **6**, 23949.

4. X. Wang, A. Jiang, T. Ho, F. Li, *Anal. Chim. Acta*, 2015, **890**, 91-97.
